# Emerging Methods for Efficient and Extensive Incorporation of Non-canonical Amino Acids Using Cell-Free Systems

**DOI:** 10.3389/fbioe.2020.00863

**Published:** 2020-07-22

**Authors:** Yang Wu, Zhaoguan Wang, Xin Qiao, Jiaojiao Li, Xiangrong Shu, Hao Qi

**Affiliations:** ^1^School of Chemical Engineering and Technology, Tianjin University, Tianjin, China; ^2^Key Laboratory of Systems Bioengineering, Ministry of Education, Tianjin University, Tianjin, China; ^3^Department of Pharmacy, Tianjin Huanhu Hospital, Tianjin, China

**Keywords:** cell-free synthetic biology, cell-free protein synthesis, non-canonical amino acids, competition elimination, *in vitro* aminoacylation

## Abstract

Cell-free protein synthesis (CFPS) has emerged as a novel protein expression platform. Especially the incorporation of non-canonical amino acids (ncAAs) has led to the development of numerous flexible methods for efficient and extensive expression of artificial proteins. Approaches were developed to eliminate the endogenous competition for ncAAs and engineer translation factors, which significantly enhanced the incorporation efficiency. Furthermore, *in vitro* aminoacylation methods can be conveniently combined with cell-free systems, extensively expanding the available ncAAs with novel and unique moieties. In this review, we summarize the recent progresses on the efficient and extensive incorporation of ncAAs by different strategies based on the elimination of competition by endogenous factors, translation factors engineering and extensive incorporation of novel ncAAs coupled with *in vitro* aminoacylation methods in CFPS. We also aim to offer new ideas to researchers working on ncAA incorporation techniques in CFPS and applications in various emerging fields.

## Introduction

Cell-free protein synthesis (CFPS) has emerged as an effective method for the production of recombinant proteins in specialty applications. By eliminating the cell membrane barrier and cell-viability constraint, CFPS offers several benefits over *in vivo* protein expression (Liu et al., 2019). Firstly, with the open nature of CFPS, almost any molecule can be manipulated precisely in the system for different research purposes, especially molecules whose incorporation is limited by inefficient transport across the cell membrane (Silverman et al., 2019). Secondly, by being able to disregard cell viability, toxic reagents and difficult to express proteins can be employed in CFPS and even some not biocompatible reaction conditions can be applied (Lu, 2017). Finally, without reproducible cells, biosafety can be guaranteed because artificial genes cannot pollute the environment through cells.

**FIGURE 1:**
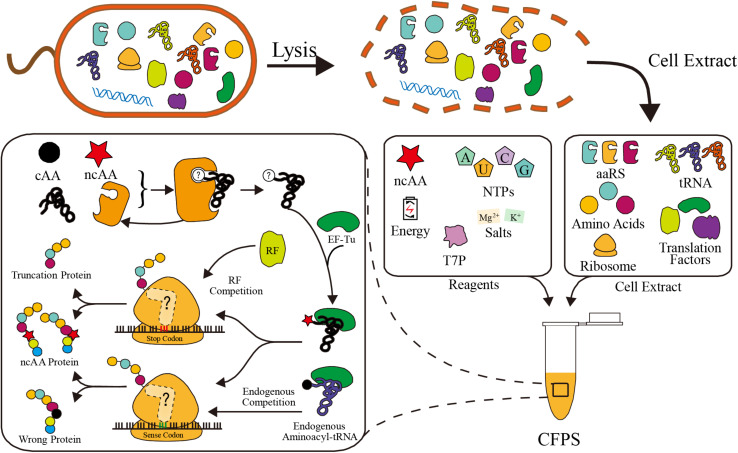
Schematic of cell extract based CFPS system preparation and competitors in ncAA incorporation. In ribosome, the peptide release factor competes with ncAA aminoacyl-tRNA in stop codon reassignment. Endogenous aminoacyl-tRNAs compete with aminoacyl-tRNAs in sense codon reassignment. In aaRS, the canonical amino acid (cAA) may compete with ncAA in aminoacylation reaction.

Basically, there are two main CFPS platforms: the PURE system (i.e., protein synthesis using purified recombinant elements), and the cell extract system. In the PURE system, all components of the transcription and translation apparatus are purified from cells individually and assembled into a well-defined CFPS system. Although all components can be defined at precise concentration, the tedious purification steps make the PURE platform much more expensive than the cell extract system (Shimizu et al., 2001). Many efforts have been made recently to reduce the costs and labor, such as one-pot purification methods and purification from fewer fusion plasmids (Wang H.H. et al., 2012; Shepherd et al., 2017; Villarreal et al., 2018; Lavickova and Maerkl, 2019). However, partial component control and modularity may be lost in these approaches. The other system relies on non-defined cell extracts. The crude cell extract is separated by lysing cells, so it contains all the native intracellular translation components. Recombinant proteins are synthesized via cell extract based CFPS with the supplementation of additives, such as energy substrates, NTPs, T7 RNA polymerase, amino acids, and salts (Dopp et al., 2019; Figure 1). Due to the simple preparation, the cell extract platform is much cheaper and convenient. Additionally, with the help of ancillary translational factors in the cell extract, this platform also has higher protein yields (Karim and Jewett, 2016). Taken together, both CFPS systems are useful platforms for different applications.

Incorporating ncAAs into proteins is an emerging biological research area with fundamental science and engineering benefits. In fundamental science, lots of questions are being answered by ncAA techniques, such as labeling proteins by isotopic or fluorescent ncAAs, and immobilization of protein using ncAAs with special side chains (Narumi et al., 2018). Post-translational protein modifications (PTM) are difficult to study due to their rapidly shifting levels in the cell. With PTM-mimicking side-chains of ncAAs, high amounts of homologous PTM proteins can be synthesized for investigation (Park et al., 2011; Rogerson et al., 2015; Kightlinger et al., 2019). In engineering applications, a growing number of artificial protein applications are also emerging, including antibody-drug conjugates (Si et al., 2016), virus-like particle drug conjugates (Bundy et al., 2008), active protein polymers (Albayrak and Swartz, 2014), and screening of artificial enzymes (Ravikumar et al., 2015).

Over 230 ncAAs have been incorporated into proteins by *in vivo* or *in vitro* methods (Gfeller et al., 2013; Dumas et al., 2015). In living cells, an orthogonal amino-acyl tRNA synthetase/tRNA (aaRS/tRNA) pair is essential to precisely incorporate ncAAs into proteins. The orthogonality means that aaRS can only incorporate ncAAs at the specific tRNA and the tRNA can only be recognized by a corresponding aaRS (Hu et al., 2014). Recently, numerous ncAA aaRS/tRNA pairs were developed based on systems from archaea. For instance, tyrosine derivatives can be installed by *Methanococcus jannaschii* TyrRS/tRNA^Tyr^ pair variants and lysine derivatives can be installed by variants of the *Methanosarcina mazei* or *Methanosarcina barkeri* PylRS/tRNA^Pyl^ (Chin, 2017). However, due to great advantages over *in vivo* research, accelerated studies are concentrating on CFPS to incorporate ncAAs. Firstly, the concentration of ncAA and aaRS/tRNA could be conveniently improved for efficient incorporation without limitation by transport across the cell membrane. Secondly, the negative components, which influence efficient ncAA incorporation, can potentially be eliminated potentially in the open CFPS environment. Thirdly, the subsequent reaction can be performed *in situ*, avoiding tedious purification from cells (Italia et al., 2019). Finally, even toxic ncAAs, which cannot be employed in living cells, could be used in CFPS combined with *in vitro* aminoacylation methods (Kawakami et al., 2008; Haruna et al., 2014; Katoh et al., 2017a). Therefore, CFPS is a novel platform for ncAA incorporation with great potential.

However, ncAA incorporation in CFPS suffers from endogenous competition (Figure 1). In order to incorporate ncAAs at precise positions, a special codon should be reassigned. Both stop codons and sense codons have been reassigned in various studies (Zimmerman et al., 2014; Schinn et al., 2017; Stech et al., 2017). There is codon competition between ncAA complexes and endogenous biomolecules, which recognize the codon as a canonical amino acid or translation stop signal, resulting in a truncated or wrong protein instead of the ncAA protein (Figure 1). Fortunately, various strategies were developed to overcome competition at different levels, fully utilizing the advantages of CFPS (Lee K.-H. et al., 2016; Cui et al., 2018; Martin et al., 2018). We summarize and discuss these efficiency improvement efforts in this review. In addition to efficient ncAA incorporation, many novel ncAAs can only be used in CFPS using *in vitro* aminoacylation methods (Ohta et al., 2008; Katoh et al., 2017b; Katoh and Suga, 2018). These novel amino acids greatly expand the scope and flexibility of CFPS ncAA incorporation, which is also gaining increasing attention.

The purpose of this review is to give an overview of the emerging methods for improving the scope and efficiency of ncAA incorporation in CFPS. We focus on strategies to raise efficiency at the competition elimination and translation factors engineering. Moreover, the novel ncAAs and *in vitro* aminoacylation methods, that can only be used in CFPS and expand the range of available ncAAs, are given special attention. This review provides a reference for researchers to choose suitable ncAA incorporation techniques for CFPS and expand the research to more potential fields.

## Strategies for Eliminating Competition

**FIGURE 2:**
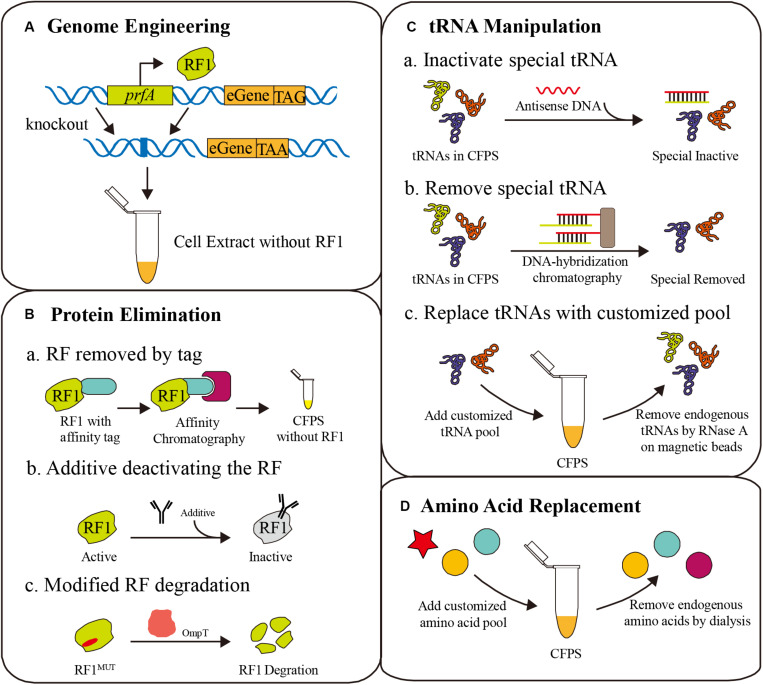
Different strategies for eliminating competition to ncAAs. **(A)** Genome engineering for deleting the prfA gene [corresponding to release factor 1 (RF1)], combined with amber codon replacement in essential genes (eGene). **(B)** Protein elimination strategies for the removal of RF1, including affinity tags, deactivating additives and protease degradation. **(C)** tRNA manipulation to erase specific tRNAs for ncAA reassignment, such as antisense DNA additives, DNA-hybridization chromatography, and tRNA pool replacement. **(D)** Amino acid replacement with a customized amino acid pool containing ncAAs.

As shown in Figure 1, the competition for codons, that seriously affects the efficiency of ncAA incorporation, always exists in codon reassignment. Competitors are classified into two categories: peptide release factors and endogenous aminoacyl-tRNA, and methods for eliminating competition include genome engineering, protein elimination, tRNA manipulation, and amino acid replacement (Figure 2). Here, we discuss the challenges and opportunities of these current and emerging methods.

### Genome Engineering

The amber codon (UAG), which is normally a translation termination signal recognized by peptide release factor 1 (RF1), is most commonly reassigned for ncAA incorporation. Deletion of the *prfA* gene encoding RF1 is a frequent strategy to improve the ncAA incorporation efficiency *in vivo* and *in vitro* (Johnson et al., 2011). Cell extracts from such strains have shown numerous advantages (Smolskaya et al., 2020). However, RF1 is essential for translation termination of over 300 genes in the genome, and the direct deletion of *prfA* could seriously affect cell growth before cell extract preparation, even leading to cell death (Chin, 2017). To address this problem, several detailed methods have been developed. These efforts and CFPS applications will be discussed in this section (Figure 2A).

The most direct method is the replacement of all amber codons with other stop codons, producing cells that do not need RF1, so that *prfA* can be knocked out without affecting cell viability. C321. ΔA is such an *E*. *coli* strain, whereby all of its 321 amber codons were replaced with ochre codons (UAA) (Lajoie et al., 2013). Without RF1 competition, ncAAs can be incorporated into proteins at high efficiency. For instance, phosphoserine was efficiently incorporated into human MEK1 kinase using CFPS based on a cell extract of C321.ΔA, in which robust production reached up to milligram quantities (Oza et al., 2015). By contrast, under RF1 competition in intracellular expression, only 25 μg of MEK1 containing phosphoserine was isolated from 1 liter of culture (Park et al., 2011). Recently, glycosylation enzymes were also successfully synthesized using this CFPS platform (Kightlinger et al., 2019). Furthermore, to improve the CFPS protein production capacity, great efforts were made to further improve C321. ΔA. Combined knockout of the genes *endA*, *gor*, *rne*, and *mazF* was shown to increase the yield of a model protein about fivefold, with up to 40 ncAAs (Martin et al., 2018). Subsequently, a C321. ΔA variant that can express T7 RNA polymerase from the chromosome was created and optimized. CFPS based on a cell extract of this strain yielded up to 70 μg/mL of a model protein with 40 ncAAs, demonstrating a highly productive one-pot CFPS system (Des Soye et al., 2019).

In addition to full replacement of amber codons, partial replacement has also been demonstrated, in which only the stop codons of some essential genes were replaced. The strains RFzero, B95.ΔA and rEc.E13.ΔA are representatives of this strategy. The RFzero strain did not contain any replaced stop codons in chromosome. Ingeniously, it was introduced the BCA7 plasmid harboring seven essential genes (*coaD*, *hda*, *hemA*, *mreC*, *murF*, *lolA*, and *lpxK*) with replaced stop codons for compensation. Subsequently, a chromosomal *prfA* deletion was carried out successfully, with only a minimal decrease of the cell growth rate (Mukai et al., 2010, 2011; Ohtake et al., 2012). Then, CFPS using a cell extract of the RFzero strain was verified to incorporate six ncAAs with high efficiency and productivity (Adachi et al., 2019). The strains B60.ΔA and B95.ΔA were developed from *E*. *coli* strain BL21(DE3) by Mukai et al. (2015) by respectively replacing 60 and 90 chromosomal amber codons before *prfA* deletion. Due to the rapid growth rates of B60.ΔA and B95.ΔA, a CFPS protocol for highly efficient incorporations of ncAAs was also described in detailed (Seki et al., 2018). Before research on CFPS based on C321.ΔA, Jewett et al., developed the RF1 deletion strain rEc.E13.ΔA, which is homologous to C321.ΔA (Hong et al., 2014, 2015). In conclusion, by bypassing the time, labor and cost needed for full codon replacement, a partial amber codon replacement strategy is a rapid and simple method for constructing the desired strains, even if the growth rate may be decreased to a certain extent. This method has been applied to diverse *E*. *coli* strains for various purposes, including MG1655, HST108, and BL21 (DE3).

In addition to the established strains for CFPS, some genome engineering strains also have considerable potential to incorporate ncAAs in CFPS. Wang et al. found that *prfA* could be deleted unconditionally in *E*. *coli* B strain derivatives. Moreover, after reverting the release factor 2 (RF2) A246T mutation back to Ala, *prfA* could also be knocked out in *E*. *coli* K12 strain derivatives, demonstrating that RF1 is non-essential in *E*. *coli* (Johnson et al., 2011, 2012; Wang, 2017). Without any stop codon replacement, it is the most rapid and simple method for RF1-free strain construction.

Although genome engineering provides a completely RF1 elimination strategy without residuals, these above methods also inevitably affect the activity and yield of CFPS and consume time and laborious for genetic manipulation. Consequently, additional strategies were developed for CFPS with unique advantages, which may be combined with genome engineering for future improvement.

### Protein Elimination

Because the open reaction environment can be controlled directly, the CFPS system is more flexible than *in vivo* translation systems. Various competition eliminating strategies are only possible in the CFPS system with its special properties. An important strategy is protein elimination from the CFPS system. By manipulating protein levels, essential proteins can remain active to aid cell growth, but can be removed from the CFPS system to remove competition. For example, RF1 can be selectively removed using affinity tags, deactivating additives, or protease degradation (Figure 2B).

Selective removal of RF1 using an affinity tag has already been demonstrated by Otting et al. This approach requires the genetic fusion of a chitin-binding domain sequence to *prfA*, so that the wild-type RF1 is replaced by a variant with three continuous chitin-binding domains at the C-terminus, which maintained the peptide release activity required for cell growth. After cell extract preparation, the lysate was filtered through a chitin column to selectively remove the tagged RF1, resulting in an improved CFPS system with efficient ncAA incorporation at multiple sites (Loscha et al., 2012; Abdelkader et al., 2015; Jabar et al., 2017).

Similarly, the RF1 protein can also be eliminated by modifying the protein to make it sensitive to certain factors and eliminating it from the CFPS system. For example, a temperature-sensitive variant of RF1 had been engineered in *E*. *coli*, and the cell extract with a thermosensitive RF1 demonstrated a 11-fold increase of efficiency in ncAA incorporation following heat treatment (Short et al., 1999). However, longstanding heat shock may result in the loss of basic activity of the CFPS system, while too little heat can leave residual RF1. Recently, another strategy was developed with no need for heat shock. Yin et al. (2017) successfully inserted an outer membrane protease (OmpT) recognition site into the switch loop of RF1. OmpT protease only identifies extracellular target proteins. Thus, the engineered RF1 can maintain the cell growth rate before cell extract preparation, after which it is cleaved by OmpT during cell lysis, which led to an increase of ncAA incorporation in CFPS.

These protein elimination strategies rely on the modification of proteins by genetic engineering. However, certain additives can also deactivate RF1 in the CFPS system. Agafonov et al. (2005) used an anti-RF1 antibody to block RF1 activity in CFPS and improve the ncAA incorporation efficiency. Nonetheless, the antibody requires expression and purification steps, which increased the costs. Consequently, the easily prepared and adaptable RNA aptamers have also been investigated for RF1 inactivation (Szkaradkiewicz et al., 2002; Shafer et al., 2004). For instance, an RNA aptamer that can orthogonally deactivate RF1 in CFPS was selected from a 50 nt random sequence RNA pool, showing the ability to increase the efficiency of ncAA incorporation (Ogawa et al., 2005; Sando et al., 2007).

RF1 could be removed at either genome or protein level. By comparison, deletion of RF1 gene from genome completely get rid of RF1 bioactivity from cell. And RF1-deficient *E*. *coli* cells have demonstrated its potential in preparation of RF1-free cell extraction for incorporation of ncAA into protein. However, knockout of gene for RF1 generated side effects, such as slow cell growth. For instance, C321.ΔA has been evolved to improve cell viability by correcting mistakes of genetic manipulation, such as off-target modifications (Wannier et al., 2018). In contrast, RF1 elimination at protein level requires complicated procedures, such as chromatography, degradation or deactivation (Agafonov et al., 2005; Sando et al., 2007; Loscha et al., 2012; Yin et al., 2017). On the other hand, without stop codon replacements by multiple-sites genome editing, protein elimination strategy can be easily applied to other strains. However, due to the open nature of CFPS, more strategies can be flexibly designed.

### Manipulation of tRNAs

When a ncAA is inserted into a protein at a sense codon, the endogenous aminoacyl-tRNA will compete with the ncAA aminoacyl-tRNA in the ribosome during translation, resulting in partially native protein. Directly manipulating tRNAs in the cell is unrealistic, but it is convenient in CFPS without a membrane barrier, freeing a large number of sense codons for ncAA incorporation by tRNA manipulation (Tajima et al., 2018). Considering the one-to-one correspondence between tRNAs and codons, artificial codon tables, and even minimum codon combinations can be achieved by tRNA manipulation in CFPS (Calles et al., 2019; Wang et al., 2020). Recently, several tRNA level methods were developed, including tRNA pool replacement, elimination using antisense oligonucleotides, and tRNA-specific enzymatic degradation (Figure 2C).

The most direct method is the replacement of all tRNAs with a tRNA pool customized according to research purposes. Due to the molecular weight of ∼25,000 with 70–90 nucleotides in tRNAs, straightforward dialysis is not capable of removing tRNA from cell extracts and some small molecular translation enzymes, such as initiation factor 1, can be lost during dialysis (Bhat et al., 2005). Nevertheless, several methods for the replacement of endogenous tRNAs have been developed. Recently, a near-complete endogenous tRNA eliminating method was reported Bundy et al. (2008) in which RNase A was attached to magnetic beads to remove native tRNAs from cell extract (Salehi et al., 2017). Additionally, semisynthetic tRNA complements, which contain the majority of 48 synthetic tRNAs, were shown to be functional in CFPS, extending the ability to customize additive tRNA pools for ncAA incorporation at multiple sense codons (Cui et al., 2015).

Considering the time-consuming steps required for native tRNA removal and custom tRNA synthesis in the tRNA pool replacement strategy, tRNA antisense oligonucleotides have emerged as a powerful tool for tRNA-specific elimination and inactivation in CFPS. Cui et al. (2017) removed the tRNA${}_{\text{CCU}}^{\text{Arg}}$ by DNA-hybridization chromatography of tRNA-specific antisense oligonucleotides, displaying a universal method for sense codon reassignment. However, without additional chromatography steps, methylated anti-tRNA oligonucleotides were demonstrated to deactivate multiple selected tRNAs by tRNA–DNA hybridization, which was induced due to heating and annealing with the tRNA mix of CFPS. This approach is more efficient than chromatography for tRNA inactivation (Cui et al., 2018).

In addition to tRNA-specific antisense oligonucleotides, the tRNA^Arg^-specific tRNase, colicin D, was also utilized for specific tRNA elimination. Colicin D specifically cleaves tRNA^Arg^ at the anticodon loop, including tRNA${}_{\text{ACG}}^{\text{Arg}}$, tRNA${}_{\text{GCG}}^{\text{Arg}}$, tRNA${}_{\text{CCG}}^{\text{Arg}}$, tRNA${}_{\text{UCG}}^{\text{Arg}}$, tRNA${}_{\text{UCU}}^{\text{Arg}}$, and tRNA${}_{\text{CCU}}^{\text{Arg}}$, which can free six codons for reassignment (Tomita et al., 2000). Kang et al. utilized resin-bound colicin D to cleave all tRNA^Arg^ in the cell-free system and supplemented synthetic tRNA${}_{\text{CCU}}^{\text{Arg}}$ for arginine translation at a single codon, successfully demonstrating the recoding of four sense codons to ncAAs (Lee K.B. et al., 2016).

These tRNA manipulation strategies are able to free a large number of sense codons for efficient ncAA incorporation which are unattainable via stop codon suppression. Nonetheless, limitations still exist. For tRNA pool replacement, only 48 synthetic tRNAs were shown functional in CFPS, and the complete synthetic tRNA substitution pool was not achieved, which may hamper some specific artificial codon tables (Cui et al., 2015). For specific tRNA elimination, the tRNA^Arg^-specific tRNase (colicin D) method was limited by tRNase deficiency and could not be adopted more widely, while the tRNA antisense oligonucleotides may be a potential powerful tool for researchers.

### Amino Acid Replacement

Due to the relaxed amino acid specificity of aaRS, a few ncAAs that are structurally similar to canonical amino acids can be charged to native tRNAs by corresponding aaRS. Examples include selenomethionine (Kigawa et al., 2002), fluorinated tryptophan analogs (Mat et al., 2010), chlorinated tyrosine analogs (Singh-Blom et al., 2014), 4-^18^F-fluoro-L-proline and 3,4-dihydroxy-l-phenylalanine (Harada et al., 2016). Thus, structurally similar ncAAs can be incorporated in place of the corresponding native amino acids. *In vivo*, a few nutrient deficient strains lacking the specific amino acid biosynthesis enzymes have been constructed and cultured with supplementation of ncAAs for artificial protein expression (Budisa et al., 2004; Montclare et al., 2009). However, ncAAs are toxic to the host strain because they will also be incorporated into essential endogenous proteins, resulting in a low growth rate and low protein yield. Moreover, residues of the corresponding canonical amino acid added during the preculture will also compete with the ncAA, leading to partially native protein.

Recently, CFPS was suggested to overcome these defects in amino acid analog incorporation. Even a detailed video protocol has been posted online (Worst et al., 2016). A cell extract of a nutrient deficient strain lacking the specific amino acid biosynthesis enzymes was prepared, followed by multiple diafiltration processes to remove all amino acids. The specific ncAA and 19 other canonical amino acids were added to the CFPS reaction for uncompetitive ncAA incorporation (Singh-Blom et al., 2014; Figure 2D). For instance, L-DOPA, a tyrosine substitute, was incorporated into protein with almost eight times higher efficiency than competitive systems (Lee K.-H. et al., 2016). The highly toxic arginine analog canavanine was also firstly incorporated into proteins in a tyrosine-replaced CFPS system (Worst et al., 2015).

This amino acid replacement CFPS method has been a cost-effective and simple alternative to ncAA incorporation methodologies without requiring engineered aaRS or tRNAs. However, owing to the loss of replaced canonical amino acids, various potential applications, which need the 20 canonical amino acids, are limited in this method. What’s more, although the specific amino acid biosynthesis enzymes had been knock out in cell-free strains, which avoided other amino acids generating the omitted amino acid to make incorrect incorporations. The omitted amino acid may still be generated from metabolic process in cell extract, such as degradation of endogenous proteins. This problem may be solved by some protease-deficient strains in future studies.

### Incorporation of Multiple Different ncAAs

In addition to the incorporation of single ncAAs, the incorporation of multiple different ncAAs into the same protein has been achieved *in vivo* and *in vitro*, widely expanding the ability to engineer artificial proteins. By repeating ncAA reassigned codons in template, it is accessible to incorporate a single type ncAA into multiple different locations without methodological modification (Johnson et al., 2011; Martin et al., 2018). On the countrary, efficiently incorporating multiple different ncAAs into a single protein requires method improvements and modifications. So this section will focus on the emerging methods of multiple different ncAAs incorporation into a single protein. However, *in vivo* methods mainly relied on stop codon repression of the cell protein expression machine, leading to an undistinguished product yield due to the competition of peptide release factors (Wan et al., 2010; Chatterjee et al., 2013a; Wang et al., 2014; Venkat et al., 2018). Only one release factor has been deleted, because the other peptide release factor must be retained for translational termination in cells. Consequently, the RF1-deficient strains, such as C321.ΔA, are currently the most appropriate host strain for reducing competition during the incorporation of multiple ncAAs (Zheng et al., 2018). Although up to three different ncAAs have been incorporated into the same protein in the C321.ΔA strain, the competition between ncAAs and peptide RF2 still had negative effects in cells (Italia et al., 2019).

Recently, cell extracts of RF1-deficient strains were also utilized in a CFPS system for efficient and modular artificial protein expression with multiple different ncAAs, but partial competition was still present, similar to the cells (Hong et al., 2014; Ozer et al., 2017; Chemla et al., 2019). Further, several total competition-removing CFPS methods were developed using different strategies. For instance, Cui et al. (2017) combined tRNA-specific affinity chromatography with stop codon repression in RF1-deficient strains for dual protein fluorescent labeling by incorporating AzF and BPFL-Cys into a single protein. This opens up the possibility of combining approaches at different levels to engineer proteins more freely.

## Translation Factors Engineering

Various factors play important role in protein translation as well, especially in ncAA incorporation. These translation factors are also engineered to facilitate efficient ncAA incorporation. In this part, engineering achievement of elongation factor Tu (EF-Tu), ribosome and aaRS/tRNA pairs are summarized and discussed.

### EF-Tu

EF-Tu is crucial in translation elongation phase, which is abundant in *E*. *coli* (Furano, 1975). EF-Tu binds aminoacyl-tRNA and delivery them to the ribosome for peptide elongation. However, EF-Tu may not efficiently bind to ncAA with macromolecular or negatively charged groups, leading to ineffective incorporation (Doi et al., 2007; Haruna et al., 2014). Thus far, many efforts had been made to improve EF-Tu in ncAA incorporation by engineering the amino acid binding domain.

With evolution of the binding domain in EF-Tu, ncAAs such as phosphoserine, phosphotyrosine, selenocysteine, pyrenylalanine, and *p*-azido-phenylalanine, were efficiently incorporated into peptide. Some ncAAs carrying negative charged phosphorylation side chain are hard to be recognized by wild type EF-Tu. Therefore, mutant EF-Tu (H67R, E216N, D217G, F219Y, T229S, and N274W) was developed to efficiently incorporated phosphoserine into peptide (Park et al., 2011) and milligram of protein with phosphoserine incorporation was obtained in cell-free system extracted from strain C321.ΔA (Oza et al., 2015). Similarly, the EF-Tu variant (E216V, D217G, and F219G) was also successfully selected for phosphotyrosine incorporation (Fan et al., 2016). And selenocysteine, another negatively charged amino acid, was also efficiently recognized by EF-Tu variant (H67Y, Q98Q, E216D, D217R, and N274R) (Haruna et al., 2014). Doi et al. (2007) developed EF-Tu mutants (E215A and D216A), in which the binding pocket of aminoacyl-tRNA was enlarged, to improve the binding of ncAAs carrying bulky side chain and this system successfully worked with ncAAs of L-1-pyrenylalanine, L-2-pyrenylalanine, and DL-2-anthraquinonylalanine as well. Incorporation of *p*-azido-phenylalanine was assisted by EF-Tu with mutant binding domain (S65A, D216A, and V274A) (Gan et al., 2017). Although the function of these EF-Tu variants has been proved *in vivo*, it is easy to applied them in CFPS by either direct addition of purified EF-Tu variants protein or co-expression with other endogenous wild-type translation factors in cell. For instance, protein with phosphoserine incorporation was synthesized to milligram in CFPS with engineered EF-Tu (Oza et al., 2015).

### Ribosome

Besides the normal proteinogenic amino acids, wild-type ribosome is also able to accept some analogs of L-α-amino acids (Dedkova and Hecht, 2019). Nonetheless, ncAAs with modified chemical backbone such as partial D-amino acids were incompatible with the wild-type translation machinery (Melnikov et al., 2019). Modification of the peptidyl transferase center on ribosomal 50S subunit has high potential to enhance special ncAAs incorporation. For instance, the mutant 23s rRNA mutations (region of 2447–2451, 2457–2462, 2057–2063, and 2502–2507) allowed efficient peptidyl transfer of D-amino acids or β-amino acids, such as D-methionine, D-phenylalanine (Dedkova et al., 2003, 2006), β-alanine and β-phenylalanine (Maini et al., 2013; Melo Czekster et al., 2016). Interestingly, mutant 16S rRNA (A238U, G849U, G1175U, G1516U) was identified as well with improved activity for selenocysteine incorporation (Thyer et al., 2013). Moreover, several novel features have been evolved in ribosome to improve ncAA incorporation. Wang et al. (2007) developed an orthogonal ribosome with a decreased functional interaction with RF1 and improved amber codon suppression. Another orthogonal ribosome was developed for efficient quadruplet codon suppression (Neumann et al., 2010). Engineered ribosome may bring the risk of losing cell viability especially under overexpression (Dedkova et al., 2003, 2006). Therefore, specially designed orthogonal ribosome may be the good solution, by which ncAA incorporation could be separated from endogenous translation and minimize the influence on cell viability.

### Evolution of aaRS/tRNA Pairs

Aminoacylation is the key procedure for orthogonality of tRNA and corresponding amino acids. Thus far, a few of specific aaRS/tRNA pairs were developed, such as the PylRS/tRNA pair for lysine, phenylalanine and pyrrolysine analogs from *Methanosarcina* spp. (Polycarpo et al., 2006; Katayama et al., 2012; Wang Y.S. et al., 2012), *Methanococcus jannaschii* and *acetivorans* TyrRS/tRNA pairs for tyrosine analogs (Ikeda-Boku et al., 2013), *Methanococcus maripaludis* SepRS/tRNA pair for phosphoserine (Rogerson et al., 2015), *Saccharomyces cerevisiae* PheRS/tRNA pair for phenylalanine and alanine analogs (Kwon and Lim, 2015), *Saccharomyces cerevisiae* TrpRS/tRNA pair for tryptophan and alanine analogs (Chatterjee et al., 2013b), *Desulfitobacterium hafniense* PylRS/tRNA for lysine analogs and *Pyrococcus horikoshii* ProRS/tRNA for proline analogs (Chatterjee et al., 2012).

However, the slow incorporation rate of nnAAs charged anminoacyl-tRNA leads to increased misincorporation of the endogenous aminoacyl-tRNA, inserting undesired canonical amino acid (O’Donoghue et al., 2012). Additionally, the site-specific incorporation requires accurate aminoacylation reaction between ncAA and its partner tRNA. Thus, directed evolution was applied to evolve efficient and accurate aaRS/tRNA pairs, including phage-assisted continuous evolution (PACE) (Esvelt et al., 2011), compartmentalized partnered replication (CPR) (Ellefson et al., 2014), parallel positive selection combined with deep sequencing and tRNA Extension (tREX) assisted with computationally identification (Zhang et al., 2017; Cervettini et al., 2020). And improved efficiency and specificity have been demonstrated for aaRS/tRNA pairs of *N*ε-Boc-L-lysine (BocK), *N*ε-acetyl-L-lysine (AcK) (Bryson et al., 2017), *p*-azido-L-phenylalanine (pAzF) (Amiram et al., 2015), 3-iodo-L-tyrosine (Oki et al., 2008). O-methyl-L-tyrosine (Cervettini et al., 2020), and so on. However, the degree of the orthogonality of these evolved aaRS/tRNA pairs is crucial for *in vivo* application. All of these evolved aaRS/tRNA pairs could also be applied in CFPS by either directly addition of purified protein or pre-expression in the cell. And, the activity of orthogonal aaRS/tRNA could be improved by accurately adjusting its relative concentration in cell-free system.

## Novel ncAAs and *in vitro* Aminoacylation Methods for CFPS

**FIGURE 3:**
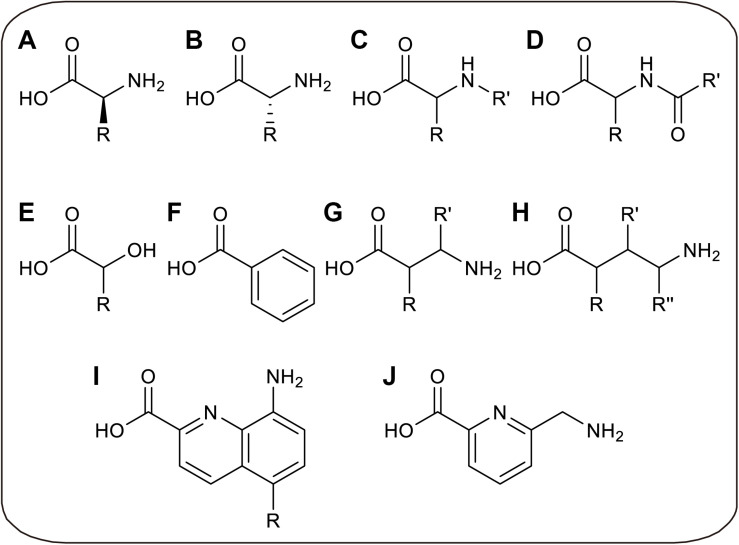
Recently developed novel ncAAs only utilized in CFPS. **(A)** L-α-amino acids with non-canonical side chains, which can only be charged to tRNAs *in vitro*. **(B)** D-α-amino acids. **(C)** N-alkylated-α-amino acids. **(D)** N-acylated-α-amino acids. **(E)** α-hydroxy acids. **(F)** α-benzoic acid. **(G)** β-amino acids. **(H)** γ-amino acids. **(I)** Quinoline-based foldamers. **(J)** Pyridine-based foldamer.

Without limitations of cell viability and membrane barrier, CFPS allows the direct addition of specific aminoacyl-tRNA to participate in ribosomal translation, in which the aminoacyl-tRNA is pre-synthesized before the CFPS reaction. By decoupling aminoacylation and translation in CFPS, several limitations can be overcome. On the one hand, because the ncAA aminoacylation reaction is physically separated from endogenous reactions, the orthogonality of aaRS is unnecessary, so that time-consuming enzyme evolution can be avoided and more ncAAs can be utilized by *in vitro* aminoacylation (Urbanek et al., 2018). On the other hand, more chemical and enzymatic methods can be utilized to generate aminoacyl-tRNAs, breaking the limitation that only analogs of L-α-amino acids can be charged by aaRS (Ninomiya et al., 2003; Murakami et al., 2006). Through *in vitro* aminoacylation methods, orthogonality requirement of aminoacylation process can be avoided, in which tRNA can only be charged by the only amino acid *in vitro*. But after translation, the released tRNA may be recharged by endogenous aaRS if it is not orthogonal to endogenous system, possibly resulting in incorrect incorporation. Thus, orthogonality evolution of aminoacylation factors can be skipped to save cost, while orthogonal tRNA is needed to achieve accurate ncAAs incorporation. Recently, several novel ncAAs were successfully incorporated into protein by such *in vitro* aminoacylation methods, including D-α-amino acids (Katoh et al., 2017b), β-amino acids (Katoh and Suga, 2018), γ-amino acids (Ohshiro et al., 2011), N-alkylated-α-amino acids (Kawakami et al., 2013), N-acylated-α-amino acids (Kwiatkowski et al., 2014), α-hydroxy acids (Ohta et al., 2008), α-benzoic acids and even foldamers (Kawakami et al., 2016; Rogers et al., 2018; Figure 3).

These novel ncAAs widely expand the ability to engineer artificial proteins with novel features, which may increase the proteolytic stability, membrane permeability, and conformational rigidity of the peptide. For instance, the thioether macrocyclic peptides incorporated with D-amino acids could provide high serum stability due to structural difference and protease resistance (Bashiruddin and Suga, 2015). And β-amino acids were considered as the potential building blocks of peptide analogs for pharmaceutical uses with the improved stability against proteolysis (Maini et al., 2013). Cyclic peptide antagonists containing N-alkylated-α-amino acids also showed more membrane-permeable and stable to proteolysis (Chatterjee et al., 2008; Baeriswyl and Heinis, 2013). The foldamers may potentially be highly water-soluble and possess cell-penetrating properties (Gillies et al., 2007). However, more applications are limited by preparation method of peptide with novel ncAAs. In this part, novel amino acids and different *in vitro* aminoacylation methods for CFPS platforms will be summarized and discussed.

### *In vitro* Aminoacylation by aaRS

**FIGURE 4:**
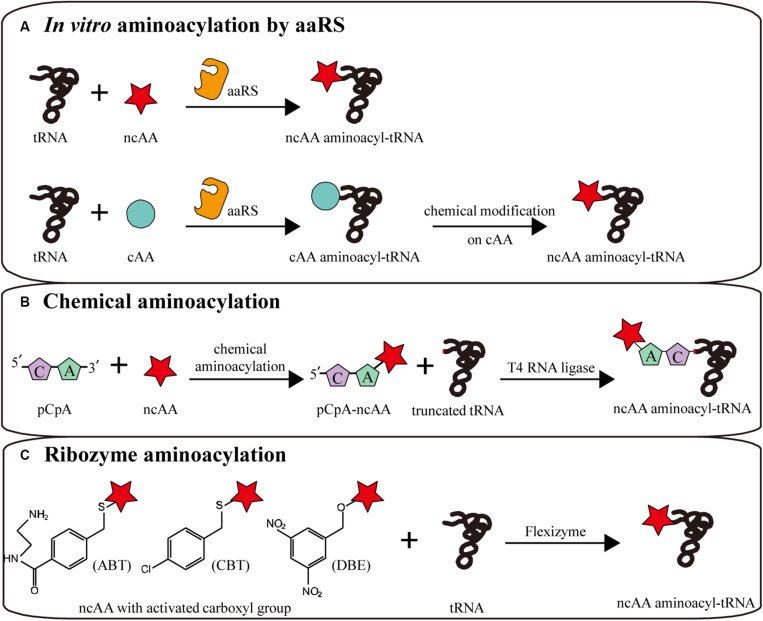
*In vitro* aminoacylation methods combined with CFPS. **(A)** *In vitro* aminoacylation by aaRS. Without an orthogonality barrier, extracellular aaRS can load tRNAs with more kinds of ncAAs. Canonical amino acid (cAA) aminoacyl-tRNAs can be chemically modified to become non-canonical. **(B)** Chemical aminoacylation. The truncated tRNA can be ligated with chemically aminoacylated pCpA-ncAA by T4 RNA ligase, generating a complete ncAA aminoacyl-tRNA. **(C)** Ribozyme aminoacylation. Flexizymes can synthesize aminoacyl-tRNAs from ncAAs with different activated carboxyl groups.

Utilizing the relaxed amino acid specificity of some aaRS, a few analogs of L-α-amino acids can be directly charged to tRNAs by aaRS (Katoh and Suga, 2019; Figure 4A). When aminoacylation and translation are conducted in the same system, in order to avoid competition between amino acids in the same aaRS/tRNA pair, the corresponding canonical amino acid should be removed to safeguard the orthogonality of the translation machine. Via pre-aminoacylation with ncAAs by aaRS, not only any codon, including stop codons, can be reassigned to a ncAA with a defined aminoacyl-tRNA, but also more efficient ncAA incorporation can be realized by optimizing additive concentrations (Urbanek et al., 2020). For instance, the general method for the expression of isotopically labeled proteins relies on aaRS charging isotopically labeled amino acids. With [^15^N,^13^C]-glutamine pre-charged by yeast glutaminyl-tRNA synthetase, homogeneous protein was obtained for high-dimensional NMR spectroscopy (Urbanek et al., 2018).

Another method is the chemical modification of side chains in canonical aminoacyl-tRNAs after normal aminoacylation by aaRS, generating ncAA aminoacyl-tRNAs to participate in translation (Figure 4A). Several side chains were successfully modified in canonical aminoacyl-tRNAs, generating moieties such as phenyllactyl amino acids (Fahnestock and Rich, 1971), N-methyl amino acids (Merryman and Green, 2004), glycosyl amino acids (Fahmi et al., 2007), fluorescence labeled amino acids and BODIPY labeled amino acids (Iijima and Hohsaka, 2009).

Compared to in vivo methods relying on introducing orthogonal aaRS/tRNA pairs into cells, in vitro aminoacylation by aaRS physically separates the ncAA aminoacylation reaction from endogenous reactions, avoiding orthogonality requirement of aaRS in ncAA aminoacylation process. The cost and time-consuming aaRS evolution can be skipped. And available ncAAs are expanded without orthogonality. However, after the dehydration condensation reaction in ribosome, the released tRNA could be recharged with unwished amino acids if it is not orthogonal to endogenous aaRS, leading to misincorporations. For precise and efficient ncAA incorporation, the corresponding tRNA needs to be orthogonal to endogenous systems. What’s more, the available specter of ncAAs is still largely limited to analogs of L-α-amino acids.

### Chemical Aminoacylation

Chemical aminoacylation is a versatile classical strategy for aminoacyl-tRNA synthesis *in vitro* (Hecht et al., 1978; Figure 4B). Although several improvements have been made, the general process remained consistent (Heckler et al., 1984; Noren et al., 1989; Ninomiya et al., 2003; Lodder et al., 2005; Kwiatkowski et al., 2014). The amino acid with appropriate protecting groups is chemically charged to the 3′-OH of pCpA or pdCpA, which was chemical synthesized. Subsequently, the corresponding truncated tRNA, lacking two bases at the 3′-terminal, is ligated with aminoacyl-pCpA (or pdCpA) by T4 RNA ligase to generate the complete aminoacyl-tRNA. In theory, this method can be used to load any tRNA with any desired amino acid, such as N-acetyl amino acids (Yamanaka et al., 2004), glycosylated serine, and tyrosine (Fahmi et al., 2007), N-methyl and N-nitro arginine (Choudhury et al., 2007), citrulline (Breuillard et al., 2015), homoarginine, and isotopically labeled canonical amino acids (Peuker et al., 2016).

In theory, the greatest strength of this method is that any desired amino acid can be charged to any tRNA. However, the combination of chemical synthesis with enzymatic ligation is labor-intensive and it is challenging to produce homogeneous aminoacyl-tRNAs. Especially the primary by-product, self-ligated tRNA, is unavoidably generated by T4 RNA ligase, leading to inefficient synthesis, which may be avoided in future researches.

### Ribozyme Aminoacylation

In addition to aaRS and chemical aminoacylation, Suga et al. evolved small ribozymes (44–46 nt) that synthesize aminoacyl-tRNAs. These so-called flexizymes can charge tRNAs with amino acids with an activated carboxyl group (Murakami et al., 2006; Goto et al., 2011). This activated carboxyl group and the 3′-terminal NCCA sequence of tRNA are crucial recognized sites of the flexizyme catalytic domain (Xiao et al., 2008). Therefore, in theory, the ncAA can be reassigned to any codon. Recently, some flexizyme variants were artificially evolved to accept amino acids with different activated carboxyl groups (Morimoto et al., 2011). The aFx, dFx, and eFx flexizymes are three widely used representatives, respectively recognizing amino acids carrying an activated amino-derivatized benzyl thioester (ABT), dinitrobenzyl ester (DBE) or chlorobenzyl ester (CBE) (Niwa et al., 2009; Passioura and Suga, 2013; Figure 4C). More recently, more than thirty activated groups were verified to be accessible to flexizyme aminoacylation, significantly expanding the range of available amino acids (Lee et al., 2019).

As an emerging method, flexizyme aminoacylation has been applied as a versatile strategy for the incorporation of a great many ncAAs. The only two limitations imposed on the amino acids are the feasibility to activate the carboxyl with a certain group and chemical stability during the aminoacylation process. In fact, different ncAAs have been utilized even with multiple incorporations, including D-α-amino acids (Katoh et al., 2017b), β-amino acids (Katoh and Suga, 2018), γ-amino acids (Ohshiro et al., 2011), α-hydroxy acids (Ohta et al., 2008), α-benzoic acids (Kawakami et al., 2016), N-alkyl-L-α-amino acids (Kawakami et al., 2013), N-methyl-L-α-amino acids (Kwiatkowski et al., 2014), and even foldamers (Rogers et al., 2018). However, the efficiency of flexizyme aminoacylation varies depending on the ncAAs with a wide range of 17–91%, which can only be optimized empirically, since there are no established design rules (Goto et al., 2011). Moreover, the ribosome incorporation capacity of ncAA aminoacyl-tRNAs also ranges from zero to high efficiency. For instance, only eight of nineteen D-α-amino acids showed highly efficient incorporation, while low efficiencies were observed in four D-α-amino acids and the other was incompatible with the ribosome (Fujino et al., 2013, 2016). Henceforth, more versatile aminoacylation and translation techniques are worth developing to satisfy the increasing demand for ncAA incorporation.

## Conclusion and Prospects

Cell-free protein synthesis has emerged as a powerful platform for engineering the genetic code with its unique advantages allowing the efficient and extensive incorporation of ncAAs, which were summarized in this review. To improve the efficiency and yield of artificial proteins, several strategies have been presented to remove ncAA competitors at different levels, including genome engineering, elimination of peptide release factors, tRNA manipulation and amino acid replacement. The pros and cons of these strategies have been discussed in detail above. By combining methods at different levels, multiple codons, including sense and nonsense codons, could be simultaneously reassigned to different ncAAs without endogenous competitors, contributing to more flexible artificial protein synthesis (Cui et al., 2017). To enhance ncAA incorporation, several translation factors have been engineered for improvement, such as EF-Tu, ribosome and aaRS/tRNA pairs. At the same time, CFPS is compatible with *in vitro* aminoacylation methods, which can supply a wider range of novel and unique ncAAs for artificial protein research. Compared with aaRS and chemical aminoacylation methods, flexizymes have been developed into a highly versatile and efficient approach for aminoacyl-tRNA synthesis, with many recent studies confirming its expansion. By emphasizing these crucial techniques for ncAA incorporation via CFPS, this review is intended to provide a bridge between current technologies and future directions in this rapidly developing field.

Looking forward, these tools have the potential to be applied in various emerging fields, such as antibody-drug conjugates, protein labeling, peptide-based materials and so on (Albayrak and Swartz, 2014; Stech et al., 2017; Suga, 2018). Especially in enzyme activity improvement, several efforts have been made with significant influence. For instance, enzyme phosphorylation is a key modification for protein activity, which may occur on tyrosines, threonines and serines. Phosphotyrosine and phosphothreonine were used to activate vasodilator-stimulated phosphoprotein (VASP) and the nicotinic acetyl choline receptor (nAChR) (Rothman et al., 2005). With phosphoserine incorporation, several homogeneous phosphorylated enzymes have been expressed, such as human MEK1 kinase, human Stimulator of Interferon Genes (STING), and Bcl2-associated agonist of cell death (BAD) (Oza et al., 2015; Zhu et al., 2019). Furthermore, methylhistidine also showed important activating effects in the enzyme catalytic center of fluorescent proteins, heme enzymes, and metalloenzymes (Xiao et al., 2014; Gan et al., 2018; Ortmayer et al., 2020). Acetyllysine and methyllysine play a crucial role in the control of chromatin and epigenetic programs by histones, which has been demonstrated by site-specific incorporation with *in vitro* aminoacylation in CFPS (Sterner and Berger, 2000; Yanagisawa et al., 2014; Wakamori et al., 2015; Xiong et al., 2016). In addition, numerous unclear modifications of amino acids in the enzymatic active center need to be investigated in the future, such as hydroxylation, glycosylation, sulfation, and amidation. Overall, this review hopes to assist researchers in choosing suitable methods to incorporate ncAAs efficiently and easily using CFPS, hopefully inspiring breakthroughs of ncAA techniques and applications in the future.

## Author Contributions

YW gathered the information, drafted the original manuscript, and designed the figures. ZW contributed to literature collection. HQ conceived the presented idea and supervised the work. All authors contributed to the article and approved the submitted version.

## Conflict of Interest

The authors declare that the research was conducted in the absence of any commercial or financial relationships that could be construed as a potential conflict of interest.
